# The potential of baicalin to enhance neuroprotection and mitochondrial function in a human neuronal cell model

**DOI:** 10.1038/s41380-024-02525-5

**Published:** 2024-03-19

**Authors:** Zoe S. J. Liu, Trang T. T. Truong, Chiara C. Bortolasci, Briana Spolding, Bruna Panizzutti, Courtney Swinton, Jee Hyun Kim, Damián Hernández, Srisaiyini Kidnapillai, Laura Gray, Michael Berk, Olivia M. Dean, Ken Walder

**Affiliations:** 1 Deakin University, IMPACT, The Institute for Mental and Physical Health and Clinical Translation, School of Medicine, Geelong, 3220 Australia; 2 Florey Institute of Neuroscience and Mental Health, Parkville, 3010 Australia

**Keywords:** Molecular biology, Genetics

## Abstract

Baicalin is a flavone glycoside derived from flowering plants belonging to the *Scutellaria* genus. Previous studies have reported baicalin’s anti-inflammatory and neuroprotective properties in rodent models, indicating the potential of baicalin in neuropsychiatric disorders where alterations in numerous processes are observed. However, the extent of baicalin’s therapeutic effects remains undetermined in a human cell model, more specifically, neuronal cells to mimic the brain environment in vitro. As a proof of concept, we treated C8-B4 cells (murine cell model) with three different doses of baicalin (0.1, 1 and 5 μM) and vehicle control (DMSO) for 24 h after liposaccharide-induced inflammation and measured the levels of TNF-α in the medium by ELISA. NT2-N cells (human neuronal-like cell model) underwent identical baicalin treatment, followed by RNA extraction, genome-wide mRNA expression profiles and gene set enrichment analysis (GSEA). We also performed neurite outgrowth assays and mitochondrial flux bioanalysis (Seahorse) in NT2-N cells. We found that in C8-B4 cells, baicalin at ≥ 1 μM exhibited anti-inflammatory effects, lowering TNF-α levels in the cell culture media. In NT2-N cells, baicalin positively affected neurite outgrowth and transcriptionally up-regulated genes in the tricarboxylic acid cycle and the glycolysis pathway. Similarly, Seahorse analysis showed increased oxygen consumption rate in baicalin-treated NT2-N cells, an indicator of enhanced mitochondrial function. Together, our findings have confirmed the neuroprotective and mitochondria enhancing effects of baicalin in human-neuronal like cells. Given the increased prominence of mitochondrial mechanisms in diverse neuropsychiatric disorders and the paucity of mitochondrial therapeutics, this suggests the potential therapeutic application of baicalin in human neuropsychiatric disorders where these processes are altered.

## Introduction

Baicalin is a flavone glycoside found in several species of the genus *Scutellaria*, flowering plants in the mint family that are commonly known as “skullcaps” [1]. It is one of the chemical ingredients of Sho-Saiko-To, a herbal supplement widely used in China and Japan to predominantly improve liver health [1, 2]. A wealth of literature suggests that baicalin may have anti-inflammatory [3], antioxidant [4, 5], neuroprotective [6, 7] and anti-cancer [8–10] effects. More recently, baicalin was also shown to improve mitochondrial function via increasing the level of adenosine triphosphate in mouse brain [11]. Remarkably, several key factors believed to contribute to human neuropsychiatric disorders include increased inflammatory cytokines and oxidative stress, which converge to drive reduced neurogenesis and mitochondrial dysfunction (for reviews see [12–15]). The above evidence strongly suggests shared underlying processes between neuropsychiatric disorders and baicalin’s mechanisms of action, and hence baicalin’s potential application in this context.

Studies have indicated baicalin’s potential for neuropsychiatric interventions. Baicalin is a positive allosteric modulator of the GABA_A_ receptor [16], and displayed antidepressant-like effects in various rodent models which was thought to be mediated by reducing inflammation, increasing neurogenesis and activating a range of relevant biological pathways such as brain-derived neurotrophic factor, extracellular signal-regulated kinase and cAMP response element-binding protein signalling [17–19]. Additionally, in the elevated plus maze, baicalin (7.5–30 mg/kg) had anxiolytic effects in mice following ischaemia [20]. In Institute of Cancer Research (ICR) mice tested using a modified Vogel conflict test, baicalin (20 mg/kg via intraperitoneal injections) also exhibited anxiolytic property without any sedative effect [21]. These cases highlight baicalin as a promising and novel agent for treatment in neuropsychiatry.

However, almost all of the preclinical research into baicalin’s therapeutic effects has been conducted in non-human systems, predominantly rodent models in vitro and in vivo. Moreover, little is known about the effects of baicalin on brain cells, with most of the previous work focusing on neuroprotective effects in ischaemia-reperfusion models (for review see [22]). These studies also often utilise strong models of stress, for instance, corticosterone injections or chronic unpredictable stress involving 24 h of a variety of stressors for many days [23, 24]. The need for understanding the effects of baicalin in human-derived brain cells under less severe stress becomes critical. Among the available in vitro models, C8-B4 (mouse microglial cell line), SH-SY5Y (human neuroblastoma cell line) and NT2-N (human neuronal-like cell line) are commonly used. Studies using SH-SY5Y cells have indicated neuroprotective and anti-apoptotic activity of baicalin [25–27]. Nevertheless, SH-SY5Y cells are known to generate pure populations of neurons following differentiation [28], raising issues regarding the representativeness of a realistic human brain environment consisting of various neuronal cell types. In contrast, NT2-N cell model has emerged as a popular alternative for human neuronal-like model as it differentiates into a mixed population of neurons, astrocytes and radial glial cells [29]. The relatively complex composition of the NT2-N cell model represents a better reflection of the human brain environment than the SH-SY5Y cell model, despite the NT2-N model taking longer (1–2 months) to culture until experimentation than SH-SY5Y cells (1–2 weeks) [28, 30].

Therefore, in the present study, we aimed to further explore baicalin’s potential therapeutic effects in NT2-N cells, a human neuronal-like cell model. As NT2-N cells are not known for their capacity to release detectable amounts of inflammatory cytokines, we used C8-B4 cells (a mouse microglial cell model) mildly stressed with lipopolysaccharide (LPS) to establish baicalin’s effects on inflammatory cytokine profiles in vitro, followed by transcriptional studies in NT2-N cells. The neuroprotective and mitochondrial enhancing properties of baicalin were demonstrated in NT2-N cells through further transcriptomics analyses, neurite outgrowth assays and mitochondrial flux bioanalysis (Seahorse assay).

## Materials and methods

### C8-B4 cell culture and treatment

C8-B4 microglial cells (ATCC® CRL-2540™) were cultured in DMEM (Life Technologies) with 10% FBS (Life Technologies) and seeded onto 24-well plates at 1.3 × 10^5^ cells/well for the measurement of cytokine release. They were stimulated with 1 ng LPS for 24 h and then received the same baicalin treatments as for NT2-N cells. Each treatment had *n* = 6 replicates and cells were tested negative for mycoplasma contamination upon purchase.

### NT2-N cell culture and treatment

NT2 human teratocarcinoma cells (ATCC® CVCL_0034™, Manassas, USA; tested negative for mycoplasma contamination upon purchase) were cultured in Dulbecco’s modified Eagle’s Medium (DMEM; Life Technologies, Melbourne, Australia) with 10% foetal bovine serum (FBS; Thermo Fisher Scientific, Melbourne, Australia), and 1% antibiotic/antimycotic solution (Life Technologies). These cells express a neuron-like phenotype following differentiation with retinoic acid [31]. The cells were treated with retinoic acid (Sigma-Aldrich, Sydney, Australia) at 1 × 10^−^^5^M for 28 days with media refreshed every 2–3 days. The neuronal markers *NeuroD* (neuronal differentiation), *GluR* (glutamate receptor) and *Tau* (cytoskeletal protein) were measured by real-time PCR (RT-PCR) to confirm the neuronal-like state of the cells (data not shown [32]). Cells were seeded onto 24-well and 96-well plates coated with 10 μg/ml poly-D-lysine (Sigma-Aldrich) and 10 μg/ml laminin (Sigma-Aldrich) at 2 × 10^5^ cells/well (24-well plates) or 8 × 10^4^ cells/well (96-well plates and 24-well XF24 cell culture microplates (Seahorse Bioscience)) with further addition of mitotic inhibitors (1 µM cytosine and 10 µM uridine; Sigma-Aldrich) for a total of seven days. The media was refreshed every 2–3 days to maintain an enriched culture of differentiated neuronal cells (NT2-N) for the drug treatments. These cells were treated with baicalin at final doses of 0.1, 1 or 5 µM, or vehicle control (i.e. dimethyl sulfoxide [DMSO]) for 24 h. Baicalin (95% purity, molecular weight = 446.36) was purchased from Sigma-Aldrich (Sydney, Australia) and the stock solution at 100 mM (22.32 mg in 500 μL of DMSO) was made up the day before the experiments. The three doses of baicalin were selected based on previously published studies utilising primate cell lines [33, 34] where baicalin displayed good cell viability (i.e. minimal cytotoxic effects) and bioactivity. The concentrations were further selected and optimised for NT2-N cells to detect gene expression levels of the above-mentioned neuronal markers. The duration of baicalin treatment (24 h) was determined based on the study by Cathcart et al. [34] where normal NT2-N cell morphology could be observed during daily monitoring of cell culture.

### Next-generation sequencing

Following the 24-h treatment, the NT2-N cells (*n* = 6 biological replicates per treatment group; sample size was determined in previous work [35]) were harvested, and RNA was extracted using RNeasy® mini kits (Qiagen, Melbourne, Australia). RNAseq libraries were prepared for all samples from 1 µg total RNA using an Illumina TruSeq RNA Sample Preparation Kit as per the manufacturer’s instructions and run on an Illumina HiSeq platform (HiSeq 2500 rapid 50bpSE; 1 flow cell) to measure genome-wide mRNA levels. The raw data were aligned to reference genomes using Bowtie 2/TopHat 2 [36]. Genes with low counts were filtered out and the data set was normalised using the weighted trimmed mean of *M*-values (TMM). The correlation of TMM normalised counts between each pair of NT2-N cell replicates is shown in Supplementary Fig. 1. The differential expression of genes between the drug treated and vehicle control groups was assessed using edgeR in R [37]. Statistical significance was corrected for multiple testing using false discovery rate (FDR) by applying the Benjamini–Hochberg method on the *p*-values. Genes with FDR *q*-values of < 0.05 were considered to be differentially expressed.

### Gene set enrichment analysis (GSEA)

GSEA was deployed using the R package clusterProfiler from Bioconductor [38] with gene lists ranked based on the sign of log fold changes (logFCs) multiplied by the log10- transformed *p*-values from the differential analysis and pathways retrieved from the Kyoto Encyclopaedia of Genes and Genomes (KEGG) database [39]. The resulting tables had enrichment scores and adjusted *p*-values calculated from 1000 permutations.

### Tumour necrosis factor (TNF)-α enzyme-linked immunosorbent assay (ELISA)

TNF-α concentrations were determined using ELISA (human Quantikine ELISA, catalogue #DTA00D, R&D Systems, Minneapolis, MN, USA). All assays were carried out following the manufacturer’s instructions.

### Neurite outgrowth assay

NT2-N cells were grown and treated with baicalin (0.1, 1 or 5 μM), a positive control (Y-27832 or Rock inhibitor for growth enhancement) or a negative vehicle control (DMSO) in black 96 well clear bottom plates (Corning®; Sigma-Aldrich, New South Wales, Australia) at 15,000 cells/well density. A total of 6 replicates derived from 6 individual clones of NT2-N cells per treatment group was used. After 24 h treatment, immunocytochemistry was performed as described previously [40]. In brief, the NT2-N cells were rinsed in ice cold PBS, and fixed with 4% paraformaldehyde (Electron Microscopy Sciences, Pennsylvania, USA) for 15 min at room temperature followed by 3 washes in PBST (PBS containing 0.1% Triton X-100). Blocking buffer (5% horse serum in PBST) was then applied and incubated for 1 h at room temperature. Monoclonal mouse antibody to β-III-tubulin (1:10,000; clone SDL.3D10; Catalogue # T8660; Sigma-Aldrich) was applied and incubated overnight at 4 °C. The next day, the cells were washed 3 times for 5 min each with PBST and treated with a biotinylated secondary horse anti-mouse antibody (1:250; Catalogue # BA-2000; Vector Laboratories, California, USA), and incubated for 1 h at room temperature. The cells were then washed 3 times for 5 min each with PBST, and streptavidin coupled Cy3 (Catalogue # S6420; Sigma-Aldrich) was added and incubated for 1 h at room temperature. Subsequently, the cells were washed once with PBST, and counterstained with DAPI (Catalogue # 62248; ThermoFisher Scientific) at a concentration of 0.1 μg/ml for 5 min. The cells were then washed and left in PBST for imaging. Images were captured using a ZOE™ Fluorescent Cell Imager (Bio-Rad, New South Wales, Australia). Five images were captured per replicate/well (middle, top, right, bottom and left), followed by analysis using the CSIRO HCA-Vision: Automated Neurite Outgrowth Analysis [41]. In brief parameters were set using one of the DMSO treated images, using the wizard guide to detect neuron body and neurites. Then all the images were processed in batch following the parameter set on step one. Ad hoc visualisation was performed on one image per well to confirm correct detection. Results for number of cells, total and average neurite outgrowth, branching layers and total and average roots were exported and analysed by statistical tests to determine if the differences between treatment groups were significant.

### Mitochondrial flux bioanalysis

The cellular bioenergetic profile of NT2-N-treated cells (each treatment group had 6 replicates or individual clones) was assessed using a Seahorse XF24 Flux Analyzer (Seahorse Bioscience, Billerica, USA). Three basal oxygen consumption rate (OCR) measurements were taken, and measurements were repeated following sequential injections of oligomycin (1 mM), carbonyl cyanide-4-(trifluoromethoxy)phenylhydrazone (FCCP; 1 mM), Rotenone (1 mM) and Antimycin A (1 mM). Basal extracellular acidification rate (ECAR) was determined from data collected at basal measurement points. The raw measurements of OCR and ECAR over time (minutes) are shown in Supplementary Fig. 2. Calculations of respiratory parameters of mitochondrial function were performed as previously described [42]. The protein concentration from each well was quantified by Pierce^TM^ BCA protein assay (Thermo Fisher Scientific) to account for differences in cell density during data analysis.

### Statistical analyses

Kolmogorov-Smirnov tests were used to check data sets for normality of distribution. Levene’s Test was used to determine whether or not equal variances could be assumed between groups. Statistical differences between all drug treatment groups were determine by Kruskal-Wallis tests. Drug treatment groups were further compared against their paired vehicle controls using independent t-tests for normally distributed data and Mann-Whitney U tests for data not normally distributed. Statistical analysis was performed using Statistical Package for the Social Sciences version 22 (SPSS) software. Differences were considered statistically significant when *p* ≤ 0.05.

## Results

**Fig. 1:**
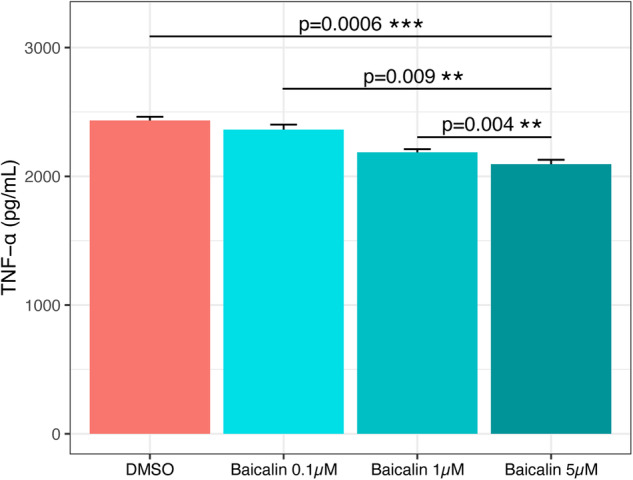
Effects of increasing doses of baicalin on TNF-α levels. C8-B4 cell culture was first stimulated with 1 ng of LPS for 24 h to induce an inflammatory response. Baicalin was then added to the C8-B4 cell culture media at final concentrations of 0.1, 1 and 5 μM and compared to vehicle control (0.01% DMSO) for 24 h. TNF-α levels (in pg/mL) were subsequently measured using ELISA. Kruskal-Wallis (between > 2 groups) and Mann-Whitney U tests (between 2 groups) were performed to test for differences between different baicalin treatment groups and *p*-values ≤ 0.05 are considered statistically significant (*p* ≤ 0.05*, *p* ≤ 0.001*** and *p* > 0.05 ns). Error bars represent standard error of the mean. DMSO Dimethyl sulfoxide, TNF tumour necrosis factor, ns not significant.

We measured the release of TNF-α into the media following treatment of LPS-stimulated C8-B4 cells with baicalin. As shown in Fig. 1, baicalin treatment was associated with a dose-related reduction in TNF-α in the media (overall *p* = 0.0006; 0.1 µM versus 1 µM *p* = 0.026; 0.1 µM versus 5 µM *p* = 0.017; 1 µM versus 5 µM *p* = 0.26). This finding aligns with previous literature indicating the anti-inflammatory properties of baicalin in murine cells.

To demonstrate that baicalin is also involved in the inflammatory pathway(s) in a human neuronal-like cell model at a transcriptional level, we extracted RNA from baicalin-treated NT2-N cells and performed GSEA. The top 20 pathways regulated by baicalin in NT2-N cells are shown in Table 1. Enriched pathways included “glycolysis/gluconeogenesis” and “oxidative phosphorylation”, suggesting effects of baicalin on bioenergetic processes, and “TNF signalling pathway” and “NF-kappa B signalling pathway”, suggesting effects on inflammatory processes. The analysis also highlighted several pathways that we have previously shown to be transcriptionally regulated in NT2-N cells following treatment with a range of psychotropic drugs including “ribosome” [43], “hippo signalling pathway” [44] and “cholesterol metabolism” [45], suggesting some mechanistic overlap between baicalin and drugs currently used to treat bipolar disorder and/or schizophrenia.

**Table 1:** Top 20 pathways transcriptionally regulated following treatment of NT2-N cells with baicalin at three concentrations.

	0.1 µM Baicalin			1 µM Baicalin			5 µM Baicalin			
Description	NES	*p*-value	p.adjust	NES	*p*-value	p.adjust	NES	*p*-value	p.adjust	sum NES
Cellular senescence	2.41	0.00019	0.0011	2.53	0.000229	0.0013	2.29	0.000224	0.0019	7.24
Ribosome	2.11	0.00019	0.0011	2.4	0.000226	0.0013	2.47	0.00022	0.0019	6.99
TNF signalling pathway	2.2	0.00019	0.0011	2.26	0.000223	0.0013	2.12	0.00022	0.0019	6.59
Lysosome	2.14	0.00019	0.0011	2.11	0.000225	0.0013	2.12	0.000222	0.0019	6.37
Focal adhesion	2.06	0.00019	0.0011	1.99	0.000232	0.0013	1.94	0.000228	0.0019	5.99
Hippo signalling pathway	2.11	0.00019	0.0011	2.02	0.000229	0.0013	1.79	0.000225	0.0019	5.93
Tight junction	1.9	0.00019	0.0011	2.01	0.000229	0.0013	1.9	0.000224	0.0019	5.81
Cholesterol metabolism	2.09	0.0002	0.0011	1.8	0.0013	0.0057	1.84	0.001304	0.0069	5.72
NF-kappa B signalling pathway	1.96	0.0002	0.0011	1.9	0.000219	0.0013	1.72	0.0013	0.0069	5.59
Apoptosis	2.09	0.00019	0.0011	1.79	0.000225	0.0013	1.67	0.0011	0.0061	5.55
C-type lectin receptor signalling pathway	1.94	0.0002	0.0011	1.82	0.000218	0.0013	1.56	0.007	0.027	5.32
Protein processing in endoplasmic reticulum	1.71	0.00019	0.0011	1.59	0.0011	0.005	1.84	0.000226	0.0019	5.15
Hematopoietic cell lineage	1.87	0.00079	0.0037	1.54	0.019	0.048	1.66	0.007797	0.029	5.07
Pyruvate metabolism	1.6	0.015	0.038	1.74	0.0028	0.0098	1.62	0.014	0.043	4.96
Amino sugar and nucleotide sugar metabolism	1.83	0.000593	0.0029	1.47	0.03	0.069	1.54	0.017	0.048	4.85
Glycolysis / Gluconeogenesis	1.64	0.0067	0.021	1.62	0.0096	0.028	1.46	0.036	0.087	4.73
Transcriptional misregulation in cancer	1.61	0.000575	0.0028	1.52	0.0025	0.0093	1.59	0.0013	0.007	4.72
Primary bile acid biosynthesis	1.65	0.011	0.03	1.28	0.17	0.26	1.73	0.0073	0.028	4.67
Oxidative phosphorylation	1.12	0.23	0.34	1.63	0.0014	0.0057	1.92	0.000223	0.0019	4.66
Breast cancer	1.74	0.000191	0.0011	1.62	0.0016	0.0065	1.29	0.059	0.13	4.65

Data are sorted by the sum of normalised enrichment scores (NES) across the 3 doses. Higher absolute value of NES indicates stronger evidence of enrichment. Positive NES indicates increased expression, while negative NES indicates decreased expression. P.adjust are *p*-values adjusted for multiple testing by applying the Benjamini–Hochberg method.

**Fig. 2:**
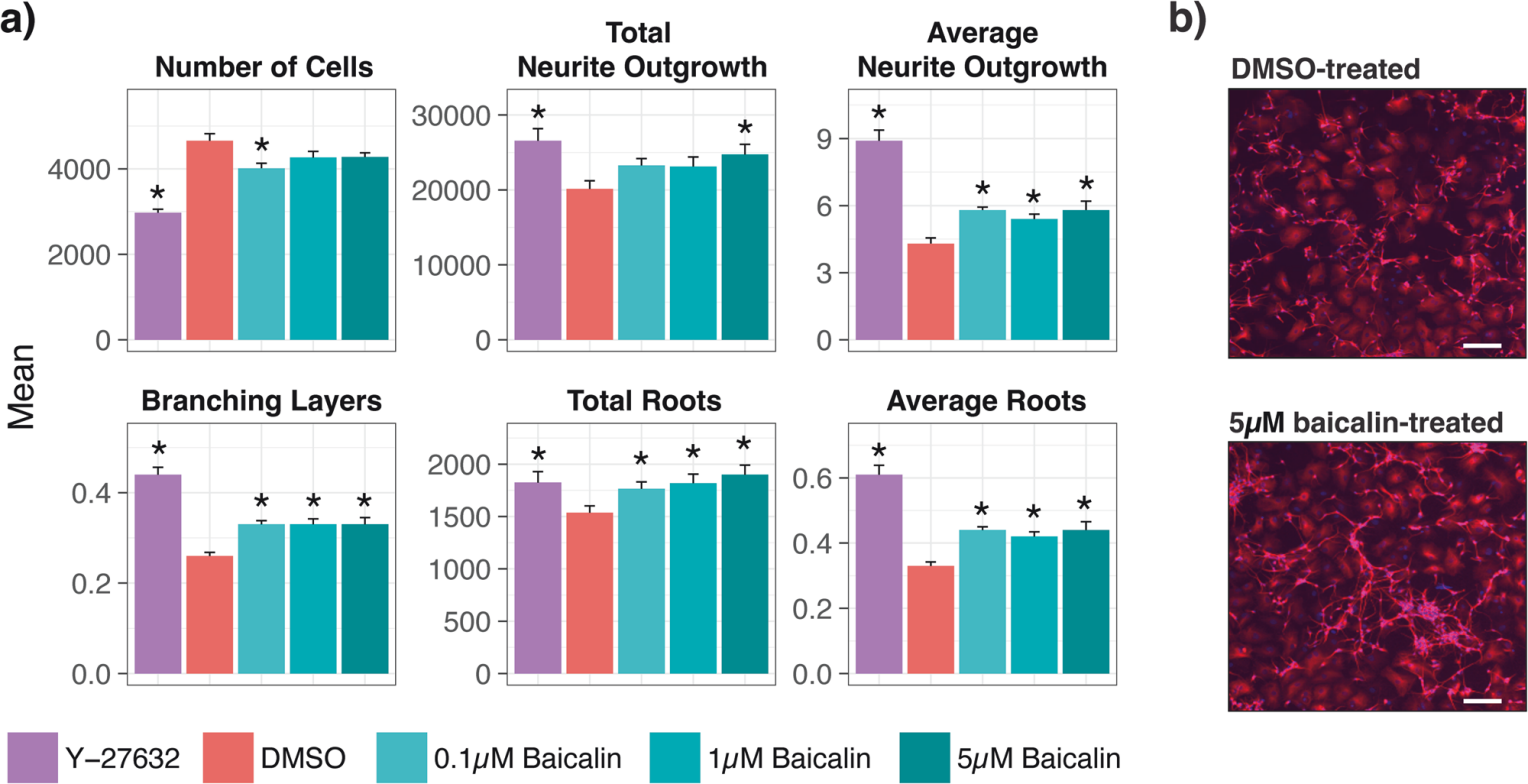
Effects of baicalin on neurite outgrowth. Baicalin at three different concentrations, positive (Y-27635) and vehicle (0.01% DMSO) controls were added to the NT2-N cell culture for 24 h before performing the neurite outgrowth assay and statistical comparisons between groups (**a**). Images were captured using a ZOE™ Fluorescent Cell Imager and five images were captured per well (**b**). Parameters were generated as previously mentioned [41]: number of cells = the number of cell bodies, neurite outgrowth = the length of main elongations stemming from the cell bodies (i.e. axons), branching layers = the highest number of times by which the original neurite is divided into a new branch, roots = the number of points at which neurites connect to the cell bodies. Independent *t* tests were performed to test for differences between individual treatment groups and vehicle controls and *p* values ≤ 0.05 are considered statistically significant (*p* ≤ 0.05*). Error bars represent standard error of the mean. Blue = nuclei, red = anti-β-tubulin III. Scale bar = 200 μm. DMSO dimethyl sulfoxide.

To further investigate the potential role of baicalin in modulating cholesterol metabolism and affecting neurite outgrowth and neuronal plasticity, we conducted neurite outgrowth assays in NT2-N cells treated with 0.1, 1 or 5 µM baicalin for 24 h. The results were compared with Y-27632 (positive control) and DMSO (vehicle control). A statistical summary of the results is shown in Supplementary Table 1. As shown in Fig. 2a, baicalin treatment was associated with increased neurite outgrowth in total length and length per cell body, number of roots in total count and per cell body, and the number of branching layers compared to vehicle control (all *p* ≤ 0.05). A representative image of the increased neurite outgrowth in NT2-N cells following baicalin treatment is shown in Fig. 2b.

**Fig. 3:**
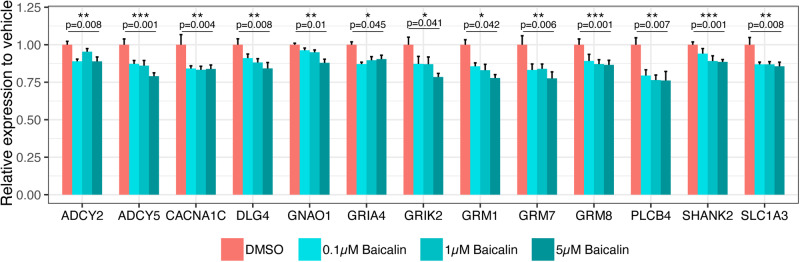
Effects of baicalin treatments on the relative expression of glutamatergic genes. NT2-N cells were treated with baicalin at three different concentrations for 24 h followed by RNA extraction. Genome-wide mRNA was measured to quantify the expression levels of genes involved in the “glutamatergic synapse” pathway relative to the vehicle control (0.01% DMSO). Kruskal-Wallis tests were performed to generate *p*-values between all treatment groups and *p*-values ≤ 0.05 are considered statistically significant (*p* ≤ 0.05*, *p* ≤ 0.01** and *p* ≤ 0.001***). Error bars represent standard error of the mean. DMSO Dimethyl sulfoxide, logFC log fold change.

Given that baicalin is known to be a positive allosteric modulator of the GABA_A_ receptor, we next investigated transcriptional effects of baicalin on the GABAergic and glutamatergic pathways following quantification of genome-wide mRNA in NT2-N cells. While baicalin had no transcriptional effect on the GABAergic pathway (data not shown), it did cause an overall down-regulation of the “glutamatergic synapse” pathway (adjusted *p* = 0.0066 by the Benjamini–Hochberg method), with consistent dose-related decreases in the gene expression of various components of the synapse (Fig. 3).

**Fig. 4:**
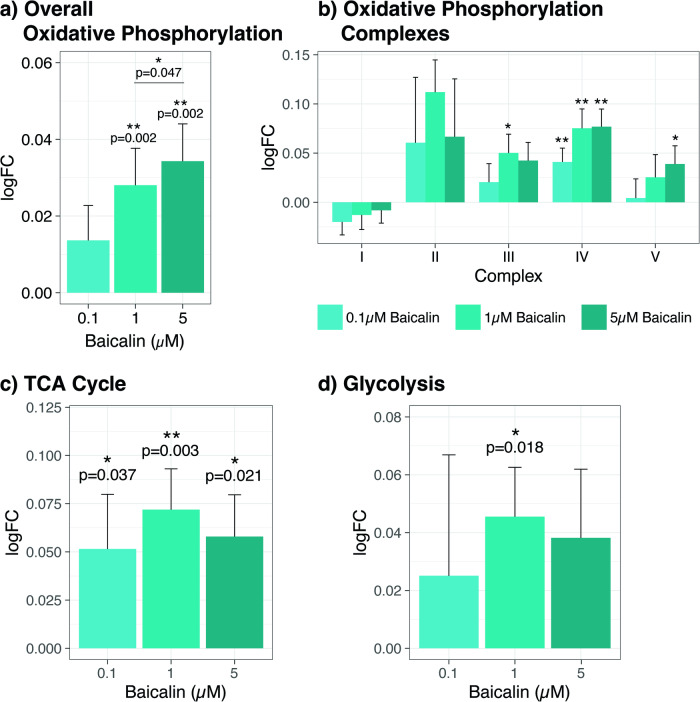
Effects of baicalin treatment on the relative expression of genes involved in oxidative phosphorylation, the TCA cycle and the glycolytic pathway. NT2-N cells were treated with baicalin at three different concentrations for 24 h followed by RNA extraction. Genome-wide mRNA was measured to quantify the relative expression levels of genes involved in **a** overall oxidative phosphorylation pathway, **b** electron transport chain complexes, **c** tricarboxylic acid (TCA) cycle and **d** glycolysis in comparison to the vehicle control (0.01% DMSO). One-sample Wilcoxon signed rank test was performed to compare individual baicalin treatment groups to respective vehicle control pairs. Mann-Whitney U test was used to compared baicalin treatment groups 1 μM and 5 μM in **a**. *P*-values ≤ 0.05 are considered statistically significant (*p* ≤ 0.05* and *p* ≤ 0.01**). Error bars represent standard error of the mean. logFC log fold change, TCA tricarboxylic acid.

We used the same approach to investigate the transcriptional effects of baicalin on oxidative phosphorylation. In Fig. 4a, baicalin treatment was associated with a dose-related increase in the expression of genes involved in the overall “oxidative phosphorylation” pathway (*p* = 0.002 for both 1 µM and 5 µM doses, 1 µM versus 5 µM *p* = 0.047) relative to vehicle (DMSO) treated cells. The electron transport chain consists of a set of molecular complexes that are essential for oxidative phosphorylation. In Fig. 4b, we showed that whilst baicalin had no transcriptional effects on genes in electron transport chain complexes I and II, it increased the relative expression of genes in complex III (1 µM dose *p* = 0.032), complex IV (0.1 µM *p* = 0.009, 1 µM *p* = 0.002, 5 µM *p* = 0.002) and complex V (5 µM *p* = 0.045). Baicalin treatment was also associated with increased transcription of genes in the tricarboxylic acid cycle at all doses (0.1 µM *p* = 0.037, 1 µM *p* = 0.003, 5 µM *p* = 0.021; Fig. 4c) and the glycolysis pathway at 1 µM (*p* = 0.018; Fig. 4d).

**Fig. 5:**
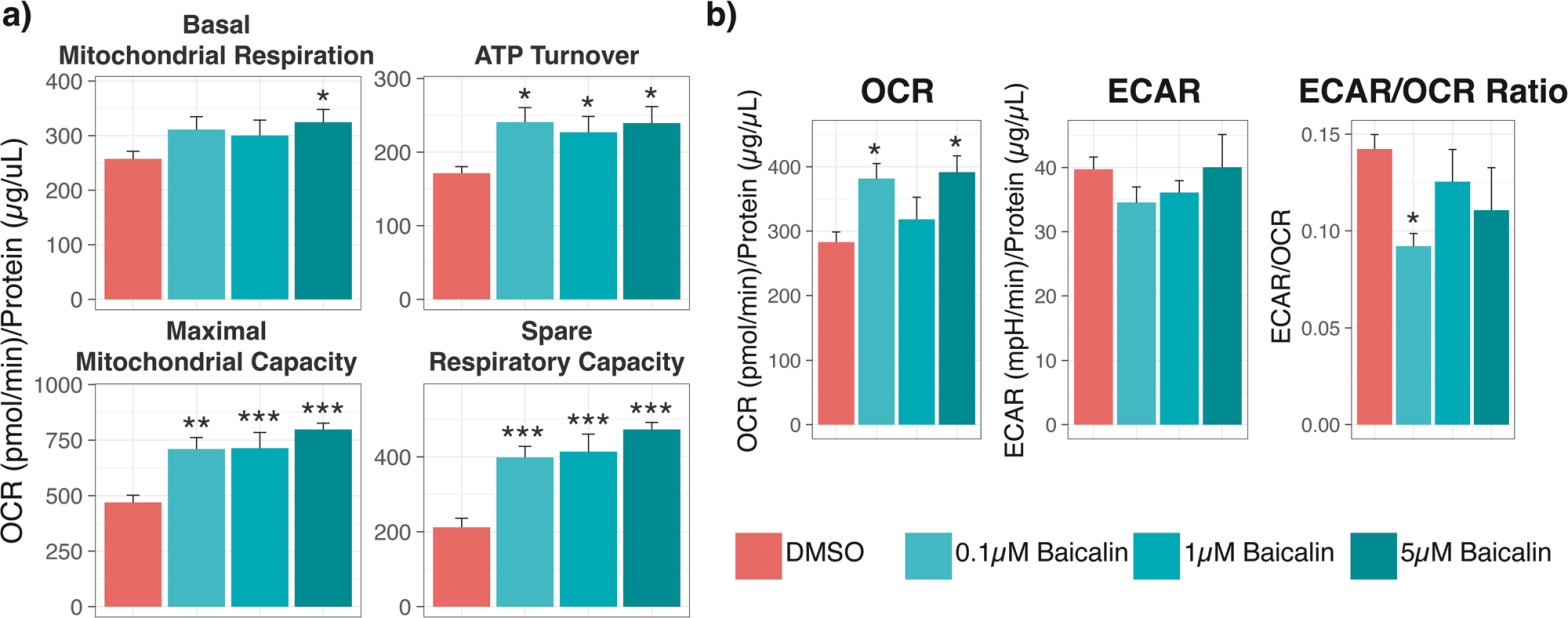
Effects of baicalin treatment on mitochondrial function. NT2-N cells (*n* = 6) were treated with baicalin at three different concentrations for 24 h followed by mitochondrial flux bioanalysis (Seahorse assay). The assay quantifies the capacity of mitochondrial respiration and overall energy metabolism through (**a**) four respiratory parameters and (**b**) oxygen consumption and extracellular acidification, respectively [72]. The rate of ATP production was measured and compared between vehicle (0.01% DMSO) and baicalin treatment groups. Kruskal–Wallis (between >2 groups) and Mann–Whitney *U*-test (between 2 groups) tests were performed to test for differences between treatment groups. *P*-values ≤ 0.05 are considered statistically significant (*p* ≤ 0.05*, *p* ≤ 0.01** and *p* ≤ 0.001***). DMSO dimethyl sulfoxide, ATP adenosine triphosphate, OCR oxygen consumption rate, ECAR extracellular acidification rate.

Mitochondrial flux bioanalysis showed that treatment of NT2-N cells with baicalin resulted in increased basal mitochondrial respiration (5 µM *p* = 0.01), ATP turnover (0.1 µM *p* = 0.01, 1 µM *p* = 0.04, 5 µM *p* = 0.01), maximal mitochondrial capacity (0.1 µM *p* = 0.004, 1 µM *p* = 0.004, 5 µM *p* = 0.0002) and spare respiratory capacity (0.1 µM *p* = 0.0006, 1 µM *p* = 0.0047, 5 µM *p* = 0.0002; Fig. 5a). Overall baicalin increased the oxygen consumption rate of the cells (0.1 µM *p* = 0.0047, 1 µM *p* = 0.0047), indicating improved mitochondrial function, but had no overall effect on the extracellular acidification rate (a measure of glycolysis; Fig. 5b).

## Discussion

In this study we aimed to elucidate the therapeutic potential of baicalin in human neuronal-like NT2-N cells. We showed in differentiated NT2-N cells that treatment with baicalin enhanced neurite outgrowth and neuronal plasticity, decreased transcription of genes in the “glutamatergic synapse” pathway and improved several markers of mitochondrial function. These results suggest that baicalin may have utility in treating neuropsychiatric diseases characterised by mitochondrial dysfunction, including depression, schizophrenia and depression in bipolar disorder.

Initially we confirmed the bioactivity of our baicalin by showing that treatment of a mouse microglial cell line (C8-B4) with baicalin after LPS stimulation resulted in reduced secretion of TNF-α into the media (Fig. 1). This is consistent with previous studies showing baicalin to have anti-inflammatory effects in vitro in a mouse macrophage cell line [46] and in a rat model of hepatotoxicity and a mouse model of non-alcoholic fatty liver disease where it reduced serum TNF-α levels [47, 48]. Furthermore, in a chronic unpredictable mild stress rat model baicalin treatment showed antidepressant-like effects on behaviour, and lowered the levels of interleuin-1β and interleukin-6 (i.e. two key pro-inflammatory cytokines) in the prefrontal cortex [49].

Numerous studies in rodents have shown that baicalin may be neuroprotective and enhance cognition (for review see [50]). One previous study reported that baicalin treatment in a repeated ischaemia-reperfusion mouse model restored dendritic spine density in the hippocampus [51], suggesting increased neuronal plasticity. However, whether such effects could be observed in human cells remains unknown. We previously showed that a bipolar disorder drug combination increased intracellular cholesterol metabolism and neurite outgrowth and hence neuronal plasticity [45]. This finding is in line with past literature showing that cholesterol metabolism plays a critical role in neurite outgrowth and synaptic function (see review [52]).

In the present study, our findings support the hypothesis that baicalin treatment can increase neuronal plasticity, and this may be mediated, at least in part, by transcriptional regulation of cholesterol metabolism, given that cholesterol is essential for the formation of new cell membranes and hence synapses [53]. Our study found evidence for co-ordinated transcriptional down-regulation of the “glutamatergic synapse” pathway, which is of interest given that baicalin is a positive allosteric modulator of the GABA_A_ receptor. In a rat ischaemia-reperfusion model, baicalin reduced proteasomal degradation of glutamine synthetase, resulting in increased glutamate disposal by astrocytes [54]. Another study in chronic unpredictable mild stress rats showed that baicalin treatment increased AMPA receptor expression in key brain structures [55], while in a rabbit model, direct injection of TNF-α into the brain induced elevations in glutamate levels in the hypothalamus, and this could be attenuated by pre-treatment with baicalin [56]. Again, we found no literature examining the effects of baicalin on the glutamate system in human cells. Our results suggest that baicalin may be protective against glutamate excitotoxicity, and this may underlie its neuroprotective effects.

Importantly, baicalin has been shown to cross blood-brain barrier in vivo, significantly strengthening baicalin’s potential application in clinical psychiatry. The blood-brain barrier is composed of mainly microvascular epithelial cells, tight junctions and immune cells. This unique composition allows the establishment of a highly selective sheath between the brain and blood circulation, playing a critical role in regulating the influx of oxygen and nutrients, efflux of waste products and protection against toxins and pathogens (for review see [57]). A structurally and/or immunologically compromised blood-brain barrier has been identified as a shared pathological feature of multiple neuropsychiatric disorders, including schizophrenia and major depressive disorder (see review [58]). Given the role of the blood-brain barrier in controlling the delivery of biological substances into the brain, the ability to penetrate the blood-brain barrier has become a prerequisite for novel pharmacological interventions for psychiatric illnesses. The fact that baicalin could pass through the blood-brain barrier was mostly consolidated in rat models. As revealed by high-performance liquid chromatography, either intravenous or intranasal administration of baicalin solution could lead to increased levels of baicalin in the periphery (i.e. plasma) and various brain regions [59, 60]. Additionally, the delivery of baicalin across the blood-brain barrier and baicalin’s half-life could be significantly enhanced through coupling with another compound [60–62]. The interaction between baicalin and the blood-brain barrier was further demonstrated by mice studies showing baicalin’s ability to restore leaky blood-brain barrier through increased expression of tight junction proteins (such as claudin-5 and ZO-1) and reduced inflammatory and oxidative stress markers [63, 64]. Together, these findings highlight the clinical potential of baicalin as a pharmacological intervention for psychiatric disorders.

Accumulating evidence suggests a prominent role for mitochondrial dysfunction and altered bioenergetics in the pathophysiology of mood disorders [65]. There has been little evidence of any effects of baicalin on mitochondrial function and bioenergetics. Numerous studies in rodents have shown protective effects of baicalin on mitochondrial-mediated apoptosis, but this can occur in the absence of changes in bioenergetics [66, 67]. In mouse macrophages, baicalin increased mitochondrial mass as well as factors regulating fission and fusion [68] and reduced mitochondrial fragmentation in a mouse model of Alzheimer’s disease [69]. Baicalin also increased mitochondrial membrane potential in an in vitro ischaemia-reperfusion model system [70] and reduced mitochondrial membrane potential in human chondrosarcoma cells [71]. Overall, there are conflicting results, very little direct measurement of mitochondrial function, and very little work in human cells. Our results show that baicalin enhanced mitochondrial function in human neuronal-like cells. Specifically, baicalin increased the transcription of genes involved in oxidative phosphorylation, the tricarboxylic acid cycle and glycolysis. Baicalin also increased basal and maximal respiration in the cells, as well as increasing ATP turnover and spare respiratory capacity. Collectively these results show a robust effect of baicalin to increase oxidative energy production in neuronal-like cells.

We acknowledge some limitations of this study. Baicalin treatment was only administered in human neuronal-like NT2 cells for 24 h, representing an acute treatment. Additionally, the baicalin treatment at doses of 0.1, 1 and 5 μM in NT2-N cell model was determined in previous studies in the laboratory. The duration of treatment and the various doses of baicalin may not achieve comparable effects on anti-inflammation, neuroprotection and mitochondrial function enhancement in experimental or clinical models of greater complexity. Utilising baicalin as a novel potential treatment or supplementation in situations of brain inflammation, such as neuropsychiatric or energy regulation diseases may require further optimisation of dosage, blood brain barrier penetrance and route of administration. Nevertheless, our study is the first to establish a complete in vitro treatment protocol with baicalin and consolidated the underlying molecular mechanisms of baicalin’s therapeutic effects in a human neuronal cell model. Longer-term baicalin treatment will be required to further validate the therapeutic effects observed in this study. Our findings will form the critical foundation from which future studies can draw inspirations for hypothesis testing and experimental design.

In conclusion, we demonstrated that 24-h in vitro baicalin treatment exhibited anti-inflammatory effects, was neuroprotective and enhanced mitochondrial function at least in part through its effects on TNF-α levels, gene expression and ATP production. This may be highly relevant to human conditions where there is impaired energy production, and/or changes in inflammation and neurogenesis, such as in bipolar depression and schizophrenia. Further investigation of the utility of baicalin as a treatment for diverse neuropsychiatric illnesses characterised by mitochondrial dysfunction is warranted.

## Supplementary information

Supplementary Table 1 Descriptive statistics for neurite outgrowth assay results per treatment group

Supplementary Figure 1 Correlations of RNA-seq count data between replicates per baicalin treatment group

Supplementary Figure 2 Raw data output of Seahorse assay

## Data Availability

Data is available upon reasonable request.
