# Supplementary figures and images for: The potential of baicalin to enhance neuroprotection and mitochondrial function in a human neuronal cell model
Source: Mol Psychiatry. 2024 Mar 19;29(8):2487–95. doi: 10.1038/s41380-024-02525-5 (PMC11412897; doi:10.1038/s41380-024-02525-5)

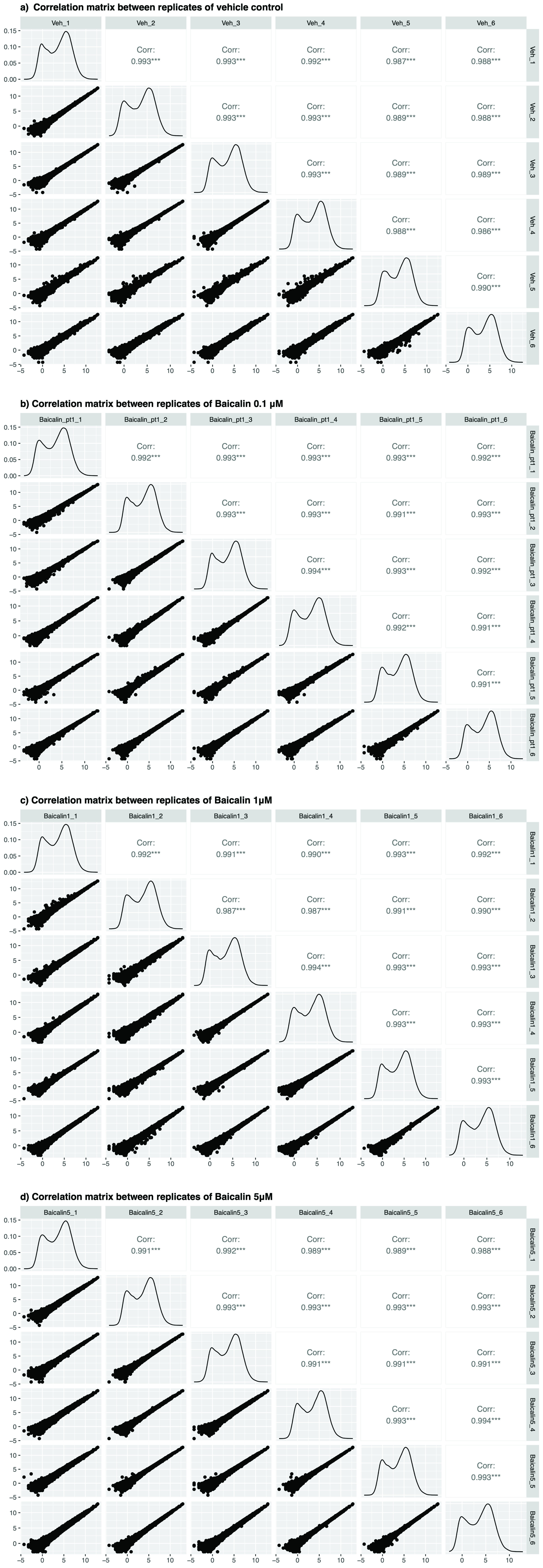

Supplement: Supplementary file 2 — Supplementary Figure 1 Correlations of RNA-seq count data between replicates per baicalin treatment group [file 41380_2024_2525_MOESM2_ESM.tif]

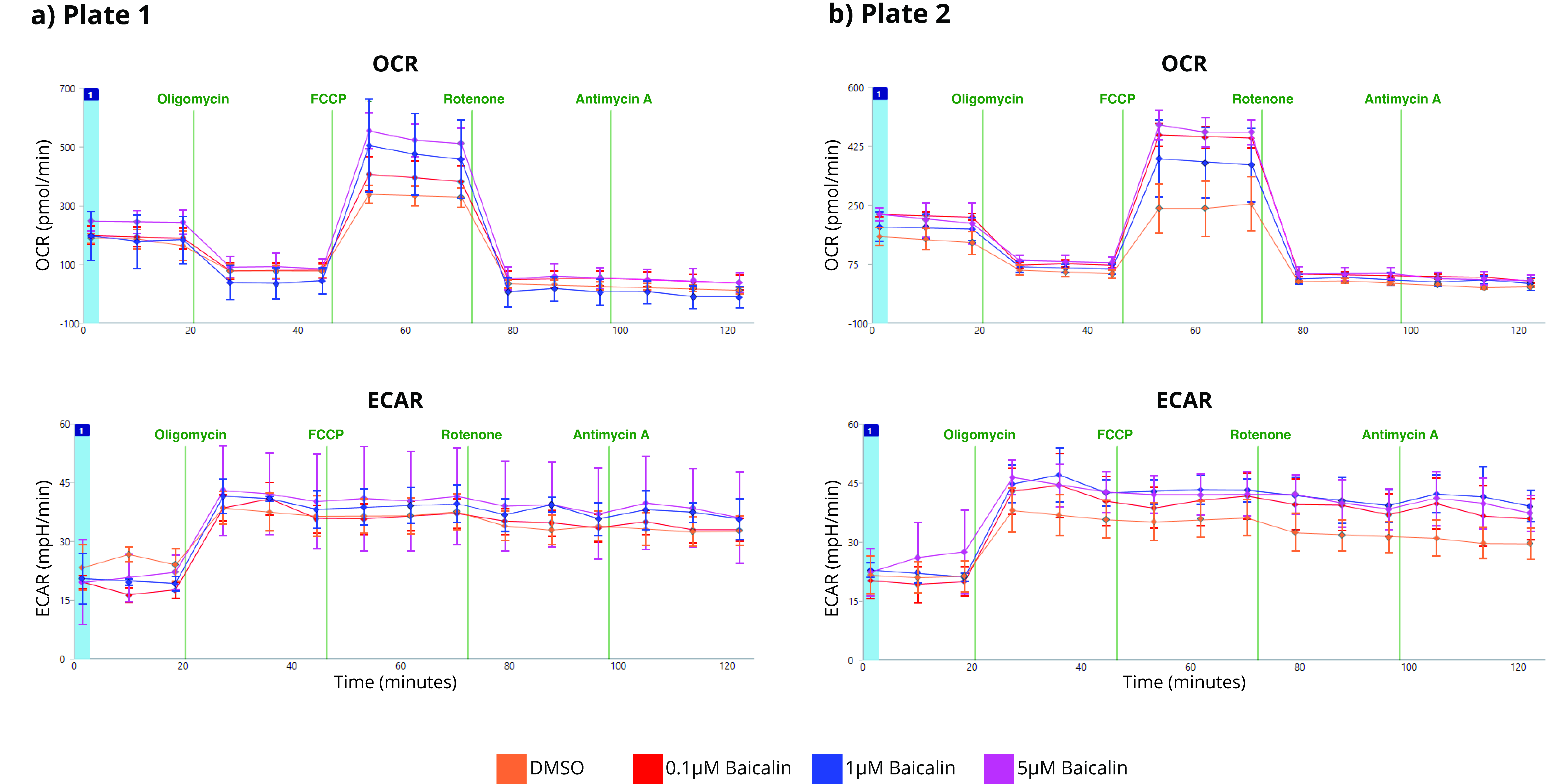

Supplement: Supplementary file 3 — Supplementary Figure 2 Raw data output of Seahorse assay [file 41380_2024_2525_MOESM3_ESM.tif]
